# HSP27 Protects Skin From Ultraviolet B -Induced Photodamage by Regulating Autophagy and Reactive Oxygen Species Production

**DOI:** 10.3389/fcell.2022.852244

**Published:** 2022-04-04

**Authors:** Zi-Yue Wang, Ang Li, Xin Huang, Gen-Long Bai, Yu-Xin Jiang, Ruo-Lin Li, Chuan Liu, Zhu-Yuan Wen, Ping Wang, Ai-Jun Chen

**Affiliations:** ^1^ Department of Dermatology, The First Affiliated Hospital of Chongqing Medical University, Chongqing, China; ^2^ Prescriptions Department, College of Traditional Chinese Medicine, Chongqing Medical University, Chongqing, China

**Keywords:** Hsp27, UVB, photodamage, autophagy, ROS

## Abstract

Ultraviolet (UV) irradiation has been well documented to be linked with almost all skin problems we know, and both dermis and epidermis may be affected to varying degrees by UV irradiation. Every time when exposed to sunlight without protection, our skin will step closer to photoaging, leading to irreversible consequences ultimately. Heat shock protein 27 (HSP27) is a vital protein involved in cell growth, autophagy, apoptosis, drug resistance, tumor genesis and metastasis. Evidence suggests that the organism is subjected to various internal and external environmental stresses (heat, oxidative stress, organic toxicants, etc.), and HSP27 with high expression has protective function. However, the expression of HSP27 in coping with UV irradiation have not been examined thoroughly. In this study, photodamage models were developed through different doses of UVB irradiation in human epidermal keratinocytes (HEKs) (30 mJ/cm^2^), human dermal fibroblasts (HDFs) (150 mJ/cm^2^) and mouse skin (2,700 mJ/cm^2^). HSP27 knockdown decreased cell viability and increased the incidence of UVB-induced reactive oxygen species (ROS) production. We got consistent results *in vivo* and vitro. Compared with that in the UVB group, the expression of LC3B was significantly lower, while the expression of p62 was significantly higher in the UVB + si-HSP27 group. It was also revealed that HSP27 knockdown reduced the expressions of some antioxidants, such as superoxide dismutase (SOD) and catalase (CAT), which accelerated UVB-induced ROS release. Moreover, histological results showed that epidermis was thickened and collagen fibers were disorganized in the UVB + si-HSP27 group. These findings have demonstrated that HSP27 might play a photoprotective role in the UVB-induced skin damage process by maintaining the normal autophagy and antioxidant level. It is implied that HSP27 could be a potential therapeutic target of photodamage. However, determination of the definitive mechanism requires further exploration.

## Introduction

Sunshine not only brings us convenience, but is also likely to endanger our health (Micka-Michalak et al., 2019). Skin, as our first and the largest line of defense in contact with the outside world, resists the adverse effects of harmful environmental factors. This is the reason why it may be hurt originally, resulting in a variety of diseases one after another. Among all the environmental factors affecting skin health, ultraviolet (UV) in sunlight is undoubtedly the one that we have the greatest exposure to. Its photodamage to skin (including cell death, photoaging, carcinogenesis, etc. (Jobe et al., 2018)) is conclusive and noteworthy. UVA (315–340 nm) and UVB (280–320 nm) are the main action spectra of skin photodamage. Although the latter is mostly absorbed by keratinocytes in epidermis due to its short wavelength, it can still reach dermis, where fibroblasts are located. The intensity of photodamage caused by UVB is 800–1,000 times that of the same dose of UVA (Syed et al., 2013).

Autophagy, a self-protection mechanism widely existing in eukaryotic cells, maintains cell homeostasis through realization of the cell metabolism (Guo et al., 2019). It plays a crucial role in the process of cell survival and death. Ultraviolet in sunlight is one of the external factors regulating autophagy (Sample & He, 2017). Although the specific mechanism is unclear, existing research shows that in terms of either keratinocytes or fibroblasts, UVB exposure can cause autophagy changes in either keratinocytes or fibroblasts (Chen et al., 2018; J. A.; Zhang et al., 2020). Inhibited autophagy may accelerate cell aging, and activated autophagy may have powerful anti-aging effect (J. Zhang et al., 2016). At the same time, some scholars believe that UVB irradiation causes photodamage by producing ROS (Cavinato & Jansen-Durr, 2017). Excessive ROS not only induces DNA damage and aggravates the possibility of carcinogenesis (Panich et al., 2016), but also disrupts the balance of oxidation/antioxidant system, impeding the production of the endogenous antioxidants such as SOD and catalase CAT, thereby reducing the skin’s ability to defend against ROS mediated photodamage (Choi et al., 2017).

HSP27 is an important member of the Small Heat Shock Proteins (sHSPs) family. In addition to its well-known molecular chaperone and anti-apoptotic effects, many studies have shown that there is a link between HSP27 and autophagy to some extent. Liu et al. (Liu et al., 2018) found that HSP27 was involved in cytoplasmic chaperone-mediated autophagy (CMA) induced by exogenous Annexin A1 (ANXA1) mimic peptide-Ac2-26 of microglia, providing a promising targeted therapeutic approach for improvement of cerebral apoplexy. Other relevant studies have proved that Phosphorylated-HSP27 (p-HSP27) is closely related to the dynamic regulation of cytoskeleton. It modulated the autophagy process by means of controlling the transport of autophagosome skeleton and the binding and degradation of lysosomes (R. Zhang et al., 2015). In another study by Liang et al. (Liang et al., 2018), it was observed that the autophagy stimulated by HSP27 promoted the anti-cancer effect of curcumin in colon cancer cells. Nonetheless, whether HSP27 can participate in the occurrence and development of UVB mediated skin photodamage by adjusting autophagy still remains unclear. The purpose of this study is to investigate the effect of HSP27 on UVB-induced acute skin photo-damage and its potential mechanism.

## Materials and Methods

### Cell Culture

All procedures were approved by the Ethics Committee of the First Affiliated Hospital of Chongqing Medical University, and all patients signed the written informed consent. HEKs and HDFs were derived from the healthy skin tissue samples of patients who underwent biopsies at the First Affiliated Hospital of Chongqing Medical University between December 2019 and May 2021. The samples cut into pieces of 0.5*0.5 cm size were isolated with 0.5% trypsin (Sigma, United States) under sterile conditions, with epidermis and dermis being separated. Digested with 0.25% trypsin (Gibco, United States) and free ethylenediamine tetracetic acid (EDTA), the epidermis cultured in the defined-keratinocyte serum-free medium (DK-SFM) was supplemented with keratinocyte growth factors (Millipore, United States) at 37°C in a 5% CO_2_ incubator. Dermis was digested by using type II collagenase (Sigma, United States) and maintained in Dulbecco’s modified Eagle medium (DMEM) (Gibco, United States) containing 10% fetal bovine serum (FBS, Biological Industries, Israel) and 1% penicillin-streptomycin (Beyotime Biotechnology, China) at 37°C in a 5% CO_2_ atmosphere. The cells grew generally from tissues after about 1 week, and were harvested when 70–80% confluency was reached. In this study, at the first two passages, keratinocytes were used, and at the second two passages, fibroblasts. The medium was altered every other day or every 3 days.

### Animals Feed

The 8-week-old C57BL/6 mice (20 ± 2 g, n = 15) were provided by the Animal Experimental Center of Chongqing Medical University. With the conditions of ambient temperature of 23 ± 2°C, relative humidity of 60%, and the alternation of day and night for 12/12 h, the mice were free to obtain food and water. All animal experiments complied with the ethical standards stipulated by the Experimental Animal Ethics Committee of the First Affiliated Hospital of Chongqing Medical University.

### UVB Irradiation

The UVB irradiation dose of cells was determined according to the results of pre-experiment, and the modeling method in animal experiments referred to the study of Sajo et al. (Sajo et al., 2017). During the entire process of the experiments, the photodamage models were established by using the UVB lamps with an emission wavelength between 290 and 320 nm (TL20/12, Philips, Netherlands). The irradiation intensity (mW/cm^2^) was measured by the UVB radiometer (Photoelectric Instrument Factory of Beijing Normal University, China), and then the irradiation time was adjusted according to the intensity.

*In vitro*, HEKs and HDFs were irradiated once at 30 mJ/cm^2^ and 150 mJ/cm^2^, respectively. Note that HDFs needed to be removed from the culture medium, washed with phosphate-buffered saline (PBS, Gibco, United States) three times, and covered with a thin layer of PBS to be irradiated. The control group was exposed to equal conditions without UVB radiation. *In vivo*, the shaved ear skin of the mice was exposed to 1,350 mJ/cm^2^ UVB radiation once a day in 2 days.

### Cell Viability Calculation

The viability of cells treated with different interventions was assessed by using the CCK-8 kit (MCE, United States) according to the manufacturer’s protocol. 100 μl of cells were inoculated in 96-well plates. After diverse treatments, 10 μl of CCK-8 solution was added to each well for sufficient reaction. The absorbance at 450 nm was measured by using Multiskan Spectrum (ThermoFisher Scientific, United States).

### An Analysis of Quantitative Reverse Transcription Polymerase Chain Reaction

24 h after UVB irradiation, extracted from cells and tissues by the RNAiso Plus Kit (TaKaRa, Japan), the total RNA was transformed into the single strand cDNA by using the RT Master Mix for qPCR (MCE, United States). Eventually, reverse transcription polymerase chain reaction was accomplished with The SYBR Green qPCR Master Mix (MCE, United States).

The primer sequences for targeting genes were listed as follows:

Cells: p62, forward: 5′-TGA​GTC​CCT​CTC​CCA​GAT​GCT-3′, reverse: 5′-GGG​GGA​TGC​TTT​GAA​TAC​TGG-3’; HSP27, forward: 5′-CCCACCCAAGTTTCCTCCT-3′,reverse: 5′-GGC​AGT​CTC​ATC​GGA​TTT​TG-3’; LC3B, forward: 5′-AAGGCGCTTACAGCTCAATG-3′,reverse: 5′-ACA​CTG​ACA​ATT​TCA​TCC​CGA-3’; CAT, forward: 5′-ACCTGTGAACTGTCCCTACCG-3′,reverse: 5′-TCA​TTG​GCA​GTG​TTG​AAT​CTC​C-3’; SOD1, forward: 5′-CGAGCAGAAGGAAAGTAATGG-3′,reverse: 5′-CCA​AGT​CTC​CAA​CAT​GCC​TC-3’; SOD2, forward: 5′-CCTGGAACCTCACATCAACG-3′,reverse: 5′-CAA​CGC​CTC​CTG​GTA​CTT​CTC-3’; ACTB, forward: 5′-AGAAAATCTGGCACCACACCT-3′,reverse: 5′-GAT​AGC​ACA​GCC​TGG​ATA​GCA-3’.

Animals: p62, forward: 5′-ACTACCCCAGAAAGTTCCAGC-3′,reverse: 5′-TTT​CCC​GAC​TCC​ATC​TGT​TC-3’; HSP27, forward: 5′-GGCATTTGGACACGGAAGT-3′,reverse: 5′-GGG​CTC​AAC​TCT​GGC​TAT​CTC-3’; LC3B, forward: 5′-GCTAACCAAGCCTTCTTCCTC-3′,reverse: 5′-TGC​TGT​CCC​GAA​TGT​CTC​C-3’; CAT, forward: 5′-CATTCAGAAGAAAGCGGTCAA-3′,reverse: 5′-TTC​TCA​GCG​TTG​TAC​TTG​TCC​AG-3’; SOD1, forward: 5′-GGGAACCATCCACTTCGAGC-3′,reverse: 5′-TCC​TGC​ACT​GGT​ACA​GCC​TTG-3’; SOD2, forward: 5′-CTGGACAAACCTGAGCCCTAAG-3′,reverse: 5′-TTG​GAC​TCC​CAC​AGA​CAC​GG-3’; GAPDH, forward: 5′-GACATCAAGAAGGTGGTGAAGC-3′,reverse: 5′-GAA​GGT​GGA​AGA​GTG​GGA​GTT-3’.

### Western Blotting Analysis

After 24 h’ exposure, proteins were isolated from cells by using RIPA lysis buffer (Beyotime Biotechnology, China) including 1% phenylmethylsulfonyl fluoride (PMSF, MCE, United States). Besides, the proteins of mice skin were extracted by T-PER™ Tissue Protein Extraction Reagent (ThermoFisher Scientific, United States). The total protein concentrations were measured by applying BCA Protein Assay Kit (Beyotime Biotechnology, China). Approximately 40 μg of every protein sample was separated on 12% sodium dodecyl sulfate polyacrylamide gel electrophoresis (SDS-PAGE) and transferred to nitrocellulose (NC) filter membranes. Then, the membranes were blocked in 7% skimmed milk in TBST for 1 h at room temperature and incubated with primary antibodies listed below at 4°C overnight: HSP27 (1:1,000, Abcam, United States), p-HSP27 (1:1,000, Cell Signaling Technology, United States), β-actin (1:2,000, Cell Signaling Technology, United States), SOD1 (1:1,000, Cell Signaling Technology, United States), p62 (1:1,000, Bimake, United States), LC3B (1:1,000, Bimake, United States), CAT (1:1,000, Bimake, United States), SOD2 (1:1,000, Bimake, United States), and GADPH (1:1,000, Bimake, United States). Washed with TBST 3 times, the membranes were then incubated with horseradish peroxidase (HRP)-linked secondary antibodies (1:5,000, Cell Signaling Technology, United States) for 1 h at room temperature. Finally, the protein bands were imaged by using the SuperSignalTM West Femto Maximum Sensitivity Substrate Kit (ThermoFisher Scientific, United States).

### siRNA Transfection

The siRNA (GenePharma, China) sequences were used to restrain the expression of cells and animals HSP27.

In all experiments, cells with considerable viability were selected to be transfected by the sequences, 5′-GGA​CGA​GCA​UGG​CUA​CAU​CTT-3' (sense strand) and 5′-GAU​GUA​GCC​AUG​CUC​GUC​CTT-3' (antisense strand). Inoculated in 6-well plates and pre-cultured for 24 h, the cells were transfected until they were completely attached to the plates. Sequentially, growth factor-free (HEKs) and serum-free (HDFs) medium containing siRNA and HiPerFect Transfection Reagent (Qiagen, Germany) were used to replace the complete medium. After 6 h of incubation, the aforesaid media were replaced with the complete medium. Subsequent experiments were performed after 24 h of culture.

The sequences exerted on animals HSP27 knockdown were 5′-UUC​UCC​GAA​CGU​GUC​ACG​UTT-3' (sense strand) and 5′-ACG​UGA​CAC​GUU​CGG​AGA​ATT-3' (antisense strand). After 1-week adaptive feed, siRNA was injected locally in one ear of the mice randomly selected. Succeeding experiments were implemented 24 h later.

### Reactive Oxygen Species Levels Detection

The cells were inoculated in 6-well plates and pre-cultured for 24 h, with the complete medium being replaced with 1 ml of growth factor-free (HEKs) and serum-free (HDFs) medium containing DCFH-DA (Beyotime Biotechnology, China). Incubated at 37°C in a 5% CO_2_ incubator for 20 min, the cells were washed with the growth factor-free (HEKs) and serum-free (HDFs) medium for three times and then irradiated with UVB lamp. After the irradiation, the labeled cells were washed twice with PBS and digested with 0.25% trypsin (Beyotime Biotechnology, China). All the samples were resuspended in 500 μl of PBS, and then analyzed by the flow cytometry and CYTExpert (Beckman Coulter, United States).

### Histological Analysis

24 h after the last irradiation, the ears of mice were cut, fixed in 4% paraformaldehyde solution for 24 h, and then were dehydrated and embedded by paraffin to section for hematoxylin-eosin (H&E) staining, Masson-trichrome staining, and immunohistochemical labeling severally. All stained skin specimens were observed under a microscope, and images were collected for subsequent analysis by ImageJ (National Institutes of Health, United States).

### Statistical Analysis

Statistical analysis was conducted by using Graphpad Prism 8.0 (Graphpad Software, Inc., United States) and IBM SPSS Statistics 22.0 (IBM Corporation, United States), and the error bars of data were shown as mean ± standard deviation (SD). The differences between the two groups were analyzed by using the Student’s t-test. With respect to more groups, one-way analysis of variance (ANOVA) was chosen to test the differences. *p* < 0.05 was considered statistically significant. All the results were obtained from at least three independent experiments.

## Results

### UVB Irradiation Inhibited Human Epidermal Keratinocytes and Human Dermal Fibroblasts Viability and Activated Autophagy

**FIGURE 1:**
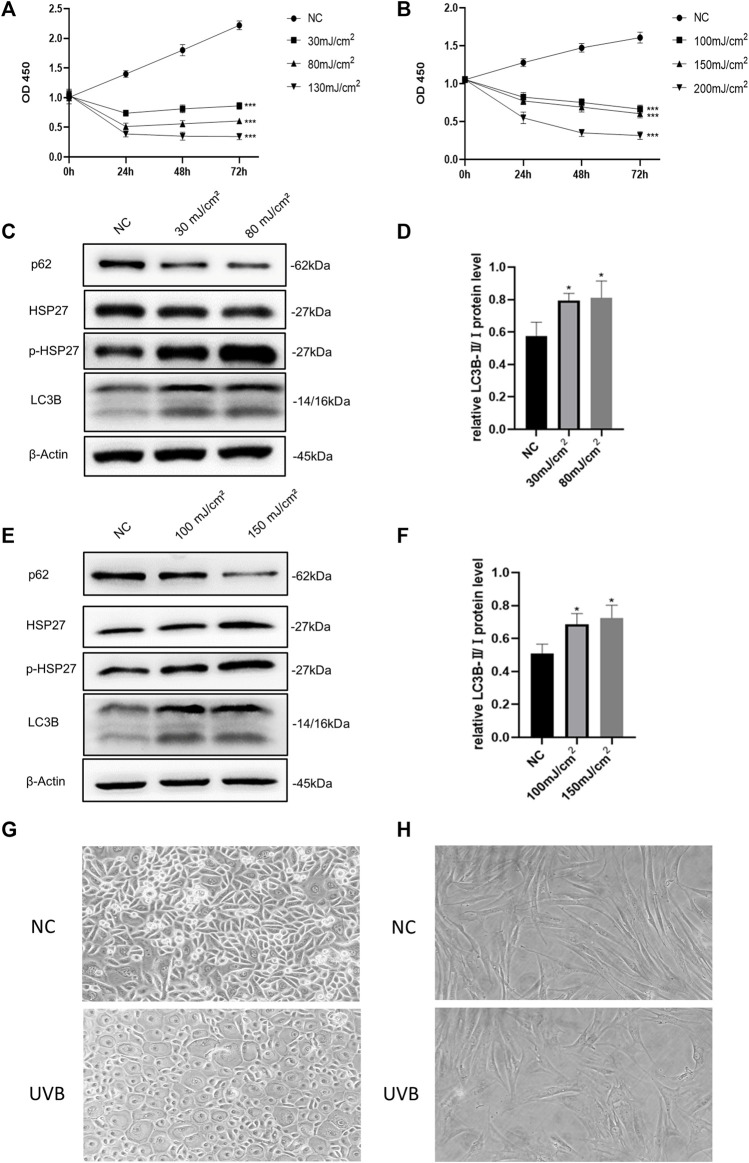
Effects of UVB irradiation on cell viability and autophagy. **(A)** The proliferation of HEKs was detected by the CCK-8 method at 0, 24, 48 and 72 h after different doses of UVB irradiation. **(B)** The proliferation of HDFs was detected by the CCK-8 method at 0, 24, 48 and 72 h after different doses of UVB irradiation. **(C)** HSP27, p-HSP27, p62, and LC3B protein expressions were detected by using Western blotting in HEKs at 24 h post-UVB, regarding β-actin as a reference. **(D)** The LC3B-Ⅱ/Ⅰ protein expressions of HEKs at 24 h post-UVB. **(E)** HSP27, p-HSP27, p62, and LC3B protein expressions were detected by using Western blotting in HDFs at 24 h post-UVB, regarding β-actin as a reference. **(F)** The LC3B-Ⅱ/Ⅰ protein expressions of HDFs at 24 h post-UVB. **(G)** Morphological variations of HEKs at 24 h after UVB irradiation was observed by microscope (200×). **(H)** Morphological variations of HDFs at 24 h after UVB irradiation was observed by microscope (200×). All the results were expressed as mean ± SD, and the experiment was repeated at least three times (*p < 0.05, **p < 0.01, ***p < 0.005).

Based on previous studies, we selected different radiation dosages to construct photodamage models of HEKs and HDFs induced by UVB irradiation (Hwang et al., 2018; Park et al., 2018). According to the results of CCK-8 detection (Figures 1A,B), UVB irradiation obviously led to a significant decrease in the activity of skin cells. When the dosages increased to 130 mJ/cm^2^ (HEKs) and 200 mJ/cm^2^ (HDFs), the cells viability decreased by more than 60%, seriously influencing the proliferation ability. Therefore, these two groups were excluded in the subsequent experiments. As shown in Figures 1C–F, compared with the control group, although the protein level of HSP27 was not significantly varied after UVB irradiation, its phosphorylated form (p-HSP27) expression was notably elevated along with the increase of dosage of radiation. Furthermore, the expression level of LC3B-Ⅱ/Ⅰ in both cells was enhanced, and the expression level of p62 was expressively decreased, indicating a probable relation between HSP27 and autophagy. Hence 30 mJ/cm^2^ (HEKs) and 150 mJ/cm^2^ (HDFs) were picked out as the radiation dosages for the development of the photodamage model of cells. Meanwhile, the morphology of both types of cells changed dramatically with UVB exposure (Figures 1G,H). HEKs altered from small polygons to larger quasi-circular ones, while HDFs became thicker, shorter and irregular in shape, confirming the success of establishment of the photodamage model.

### Heat Shock Protein 27 Knockdown Hindered UVB Irradiation and Induced Autophagy of Human Epidermal Keratinocytes and Human Dermal Fibroblasts

**FIGURE 2:**
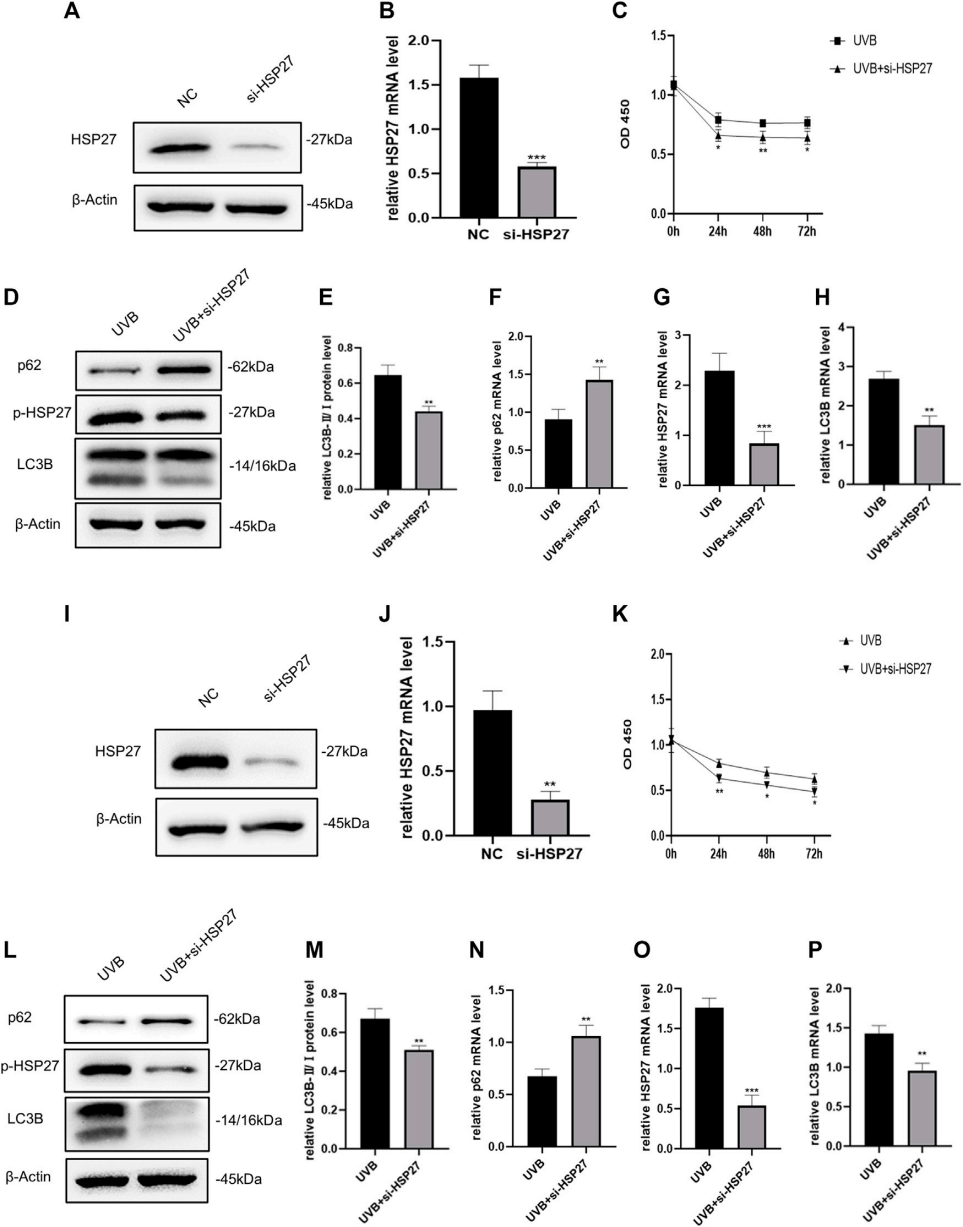
Effects of HSP27 knockdown on cell activity and autophagy under UVB irradiation. **(A,D)** HSP27, p-HSP27, p62 and LC3B protein expressions were detected by using Western blotting in HEKs after HSP27 gene knockdown, regarding β-actin as a reference. **(B,F**–**H)** HSP27, p62 and LC3B mRNA levels were detected by using RT-qPCR in HEKs after HSP27 gene knockdown, quantified by using ACTB as a reference. **(C)** The proliferation ability of HEKs was detected by the CCK-8 method at 0, 24, 48 and 72 h after UVB irradiation in the control group and the si-HSP27 group. **(E)** The LC3B-Ⅱ/Ⅰ protein expressions of HEKs after HSP27 gene knockdown. **(I,L)** HSP27, p-HSP27, p62 and LC3B protein expressions were detected by using Western blotting in HDFs after HSP27 gene knockdown, regarding β-actin as a reference. **(J,N**–**P)** HSP27, p62 and LC3B mRNA levels were detected by using RT-qPCR in HDFs after HSP27 gene knockdown, quantified by using ACTB as a reference. **(K)** The proliferation ability of HDFs was detected by the CCK-8 method at 0, 24, 48 and 72 h after UVB irradiation in the control group and the si-HSP27 group. **(M)** The LC3B-II/I protein expressions of HDFs after HSP27 gene knockdown. All the results were expressed as mean ± SD, and the experiment was repeated at least three times (*p < 0.05, **p < 0.01, ***p < 0.005).

To explore the effect of HSP27 on UVB-induced photodamage, we successfully constructed HSP27 low expression models by means of siRNA (Figures 2A,B,I,J). The proliferation ability of cells was evaluated by the CCK-8 test, showing that the growth of UVB + si-HSP27 group was significantly inhibited, compared with the group of cells irradiated by UVB alone (Figures 2C,K). We also found that after HSP27 gene knockdown, the protein or mRNA expression level of HEKs and HDFs exhibited p62 accumulation and LC3B-Ⅱ/Ⅰ reduction (Figures 2D–H,L–P). These results suggested that HSP27 might act a photoprotective part in UVB induced photodamage by promoting autophagy.

### Heat Shock Protein 27 Knockdown Exacerbated UVB-Induced Reactive Oxygen Species Amassing and Attenuated the Antioxidant Capacity of Human Epidermal Keratinocytes and Human Dermal Fibroblasts

**FIGURE 3:**
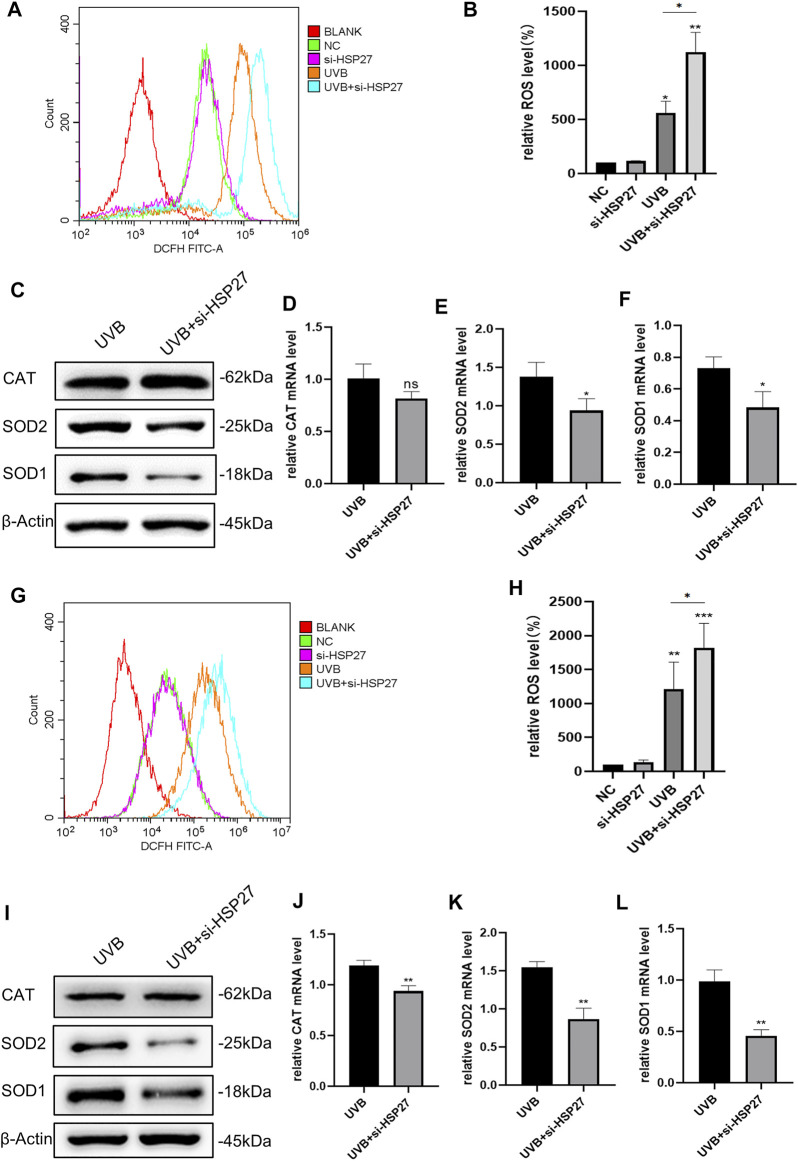
Effect of HSP27 knockdown on the antioxidant capacity of cells irradiated with UVB. **(A,B)** Alterations of ROS level in HEKs after HSP27 gene knockdown. **(G,H)** Alterations of ROS level in HDFs after HSP27 gene knockdown. **(C)** CAT, SOD1 and SOD2 protein expressions were detected by using Western blotting in HEKs after HSP27 gene knockdown, regarding β-actin as a reference. **(I)** CAT, SOD1 and SOD2 protein expressions were detected by using Western blotting in HDFs after HSP27 gene knockdown, regarding β-actin as a reference. **(D–F)** CAT, SOD1, SOD2 mRNA levels were detected by using RT-qPCR in HEKs after HSP27 gene knockdown, quantified by using ACTB as a reference. **(J–L)** CAT, SOD1, SOD2 mRNA levels were detected by using RT-qPCR in HDFs after HSP27 gene knockdown, quantified by using ACTB as a reference. All the results were expressed as mean ± SD, and the experiment was repeated for at least three times (*p < 0.05, **p < 0.01, ***p < 0.005).

In an animal experiment related to cardiac senescence, researchers found that specific motivation of the myocardial HSP27 expression could resist the cardiac functional damage caused by senescence, interfering the production of ROS in cells (Lin et al., 2016). For the sake of determining whether this phenomenon existed in the skin photodamage induced by UVB irradiation, we firstly contrasted the ROS levels before and after UVB irradiation. As illustrated in Figures 3A,B,G,H, UVB irradiation could distinctly attract the production of ROS in HEKs and HDFs. In addition, in the UVB + si-HSP27 group, the ROS level was aggrandized even more. Then, we probed the variations of some intracellular antioxidants. Unscrambling the western blotting and RT-qPCR outcomes (Figures 3C–F,I–L), the production of CAT, SOD1 and SOD2 were lessened in post-irradiation cells with inhibited HSP27, compared to the mere UVB irradiation group. CAT mainly decreased at mRNA level of HDFs while SOD decreased at both protein and mRNA levels of these 2 cells, demonstrating that the latter was more likely to weaken the antioxidant state of cells. Therefore, we speculated that HSP27 might also have a photoprotective impact on cells by suppressing UVB-induced oxidative stress response and ROS production.

### Heat Shock Protein 27 Knockdown Aggravated Skin Photodamage Treated by UVB in Mice

**FIGURE 4:**
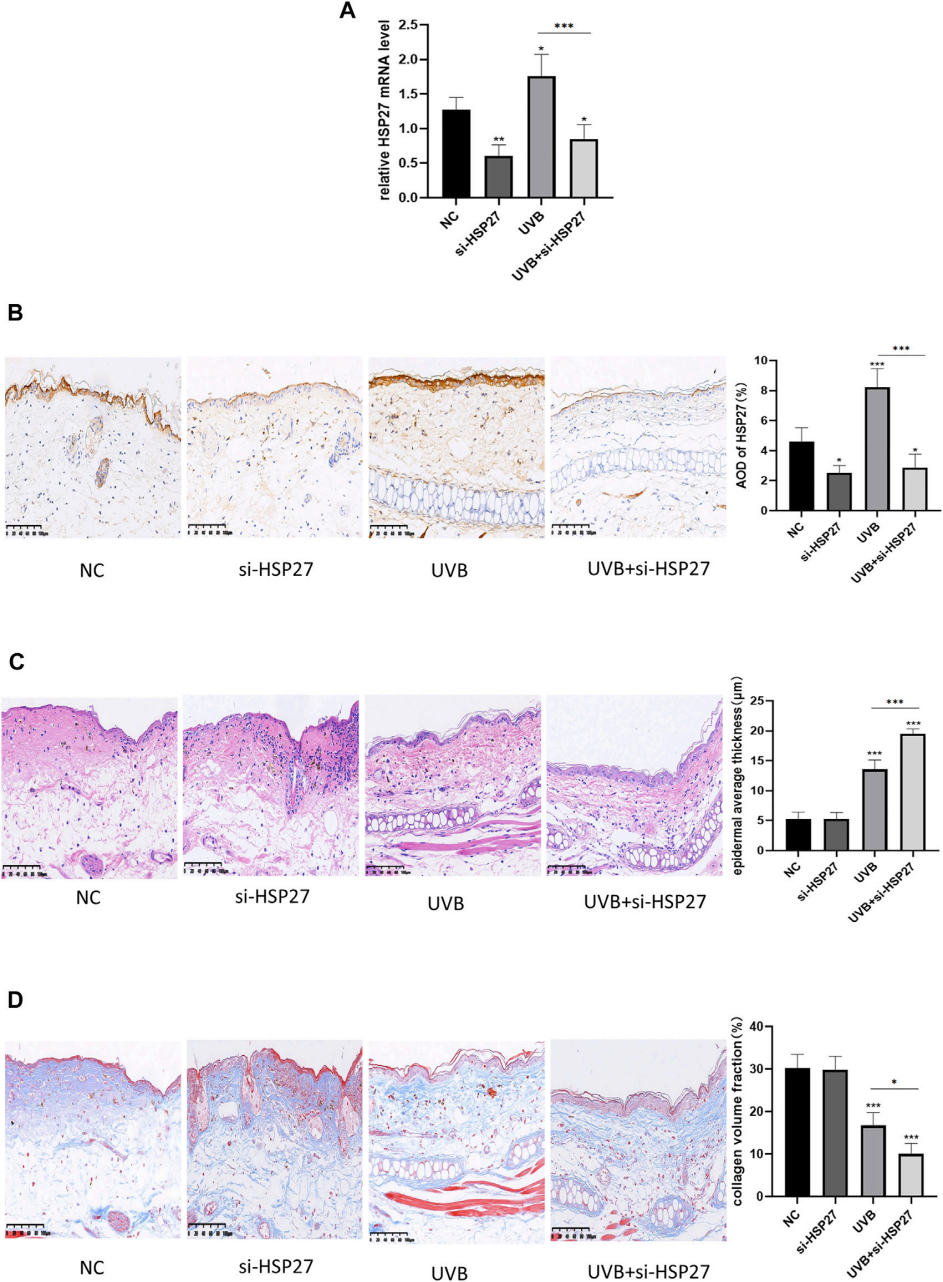
Effects of HSP27 knockdown on UVB-induced skin pathological changes in mice. **(A)** HSP27 mRNA level was detected by using RT-qPCR after HSP27 gene knockdown, quantified by using GAPDH as a reference. **(B)** Immunohistochemical marker for HSP27 results and quantification of Average Optical Density (AOD). **(C)** H&E staining results and quantification of epidermal average thickness. **(D)** Masson-trichrome staining results and quantification of collagen volume fraction. All the results were expressed as mean ± SD, and the experiment was repeated at least three times (*p < 0.05, **p < 0.01, ***p < 0.005).

In animal experiments, the expression of HSP27 was increased in the tissues with UVB exposure, and siRNA was also applied to specifically down-regulate the expression of HSP27 in mice ear skin (Figures 4A,B). Studies illustrated that epidermal incrassation and differentiation in connective tissue were prevalent in photodamage skin (Tak et al., 2020). H&E and Masson-trichrome staining were used to assess the histopathologic changes of mice skin after 24 h of the last UVB irradiation. As presented in Figures 4C,D, compared with those in the non-irradiated groups, the epidermis was thickened, cells were disordered and collagen fibers were loose, reduced, fractured and disorganized in the irradiated group. Apart from that, the above changes were more remarkable in the UVB + si-HSP27 group than those in the UVB group.

### Heat Shock Protein 27 Knockdown Alleviated the Autophagy and Antioxidant Capacity of Mice Skin Treated by UVB Irradiation

**FIGURE 5:**
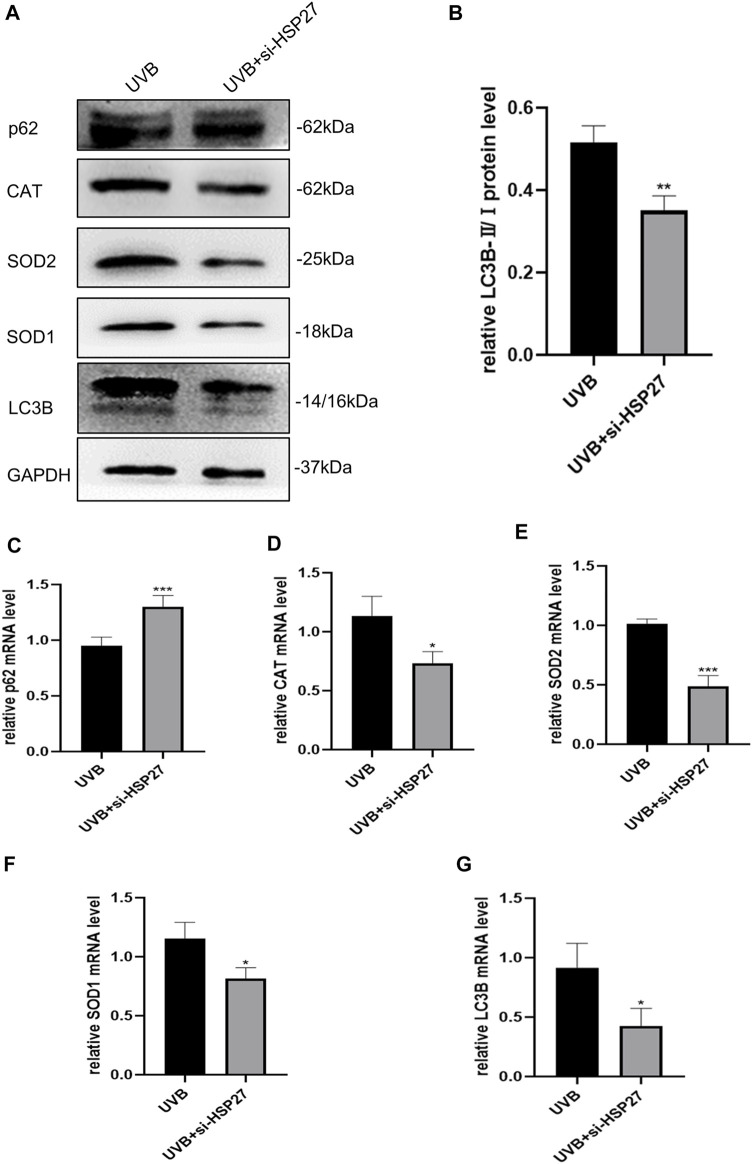
Effects of HSP27 knockdown on skin autophagy and antioxidant capacity of mice irradiated with UVB. **(A)** p62, LC3B, CAT, SOD1 and SOD2 protein expressions were detected by using Western blotting after HSP27 gene knockdown, regarding GAPDH as a reference. **(B)** The LC3B-II/I protein expressions after HSP27 gene knockdown. **(C–G)** p62, LC3B, CAT, SOD1 and SOD2 mRNA levels were detected by using RT-qPCR after HSP27 gene knockdown, quantified by using GAPDH as a reference. All the results were expressed as mean ± SD, and the experiment was repeated at least three times (*p < 0.05, **p < 0.01, ***p < 0.005).

In order to determine whether the role of HSP27 in UVB-induced photodamage was consistent *in vivo* and vitro experiments, the autophagy related indexes p62 and LC3B and antioxidant indexes CAT, SOD1 and SOD2 of mice skin tissues were also examined. Fortunately, similar results were acquired in both protein levels (Figures 5A,B) and mRNA levels (Figures 5C–G). The mice co-treated with UVB irradiation and HSP27 downregulation manifested an apparent increase of p62, while visible decrease in the expressions of LC3B, CAT, SOD1 and SOD2, compared to the UVB group. This further revealed that HSP27 was perhaps beneficial to preventing skin from UVB-caused photodamage by regulating autophagy level and antioxidant capacity of the organism.

## Discussion

It is well known that either acute exposure or chronic repeated exposure to UV light will lead to various skin lesions, such as sunburn, chronic actinic dermatitis, and even induce carcinogenesis (Sharma et al., 2018). At the same time, the absorption of radiation by skin chromophores or the formation of ROS can give rise to premature skin senescence, pigmentation and collagen loss (Silva et al., 2017). Due to the differences in the degree of clinical manifestations and the recovery speed of the body, most researchers pay more attention to the chronic photodamage caused by UV light, especially the consequences of photoaging (Zhao et al., 2018; Rui et al., 2019; Yang et al., 2019). However, the progress in preventing the acute photodamage induced by UV radiation is also required to be explored. Although UVB is mainly absorbed by keratinocytes in the epidermis, it can still reach the upper dermis, and generate pathological variations of the dermis through signaling pathways (Takeuchi & Runger, 2013). On the grounds of reviewed literature and previous studies of our team, we initially irradiated HEKs with 30 mJ/cm^2^, 80 mJ/cm^2^ and 130 mJ/cm^2^, and HDFs with 100 mJ/cm^2^, 150 mJ/cm^2^ and 200 mJ/cm^2^. Subsequently, considering the diversification in cell activity, autophagy activity and HSP27 expression level, a new UVB-induced photodamage model *in vitro* was proposed.

As a molecular chaperone, HSP27 can assist in protein folding, aggregation, transportation and stabilization. Beyond that, it is capable of restraining apoptosis through caspase-dependent and caspase-independent pathways (Acunzo et al., 2012). Given previous outcomes of our group, we revealed that rapamycin, an autophagy activator, might potentially to preserve skin fibroblasts from UVA-induced photoaging by inhibition of p53 and p-HSP27 and counteract the collagen destruction operated by HSP27 knockdown (Bai et al., 2021). Interestingly, in our trials, p-HSP27 expression modulation synchronizing with the autophagy activation was discovered in either photodamage models of HEKs and HDFs *in vitro* or these models of C57BL/6 mice *in vivo*. In addition, HSP27 knockdown would exacerbate the destruction of collagen resulting from UVB exposure. These phenomena manifested that HSP27 might protect the skin from photodamage caused by UVB irradiation by promoting autophagy.

UVB exposure will destroy the antioxidant defense system, resulting in oxidative stress (Wang et al., 2016). A large number of ROS, such as superoxide anion radical (·O_2_-), hydrogen peroxide (H_2_O_2_) and hydroxy radical (-OH), etc. are generated in the cell (Di Meo et al., 2016). The ROS amount produced by keratinocytes and fibroblasts is related to UV radiation in a dose-dependent manner. Oxidative stress reaction induced by ROS generates lipid peroxidation and DNA injury, resulting in photodamage to the skin (Hideg et al., 2013). Other studies proved that HSP27 had a certain antioxidant capacity. For example, VB-037, a quinoline derivative, restrained ROS level by controlling the HSP27 and P38/JNK signaling pathways to improve neuron damage and neuroinflammation, providing a promising direction for exploring therapeutic candidates for Alzheimer’s disease (Chiu et al., 2019). In addition, based on analysis of clinical samples from coronary artery disease (CHD) patients and the atherosclerosis mice models, researchers detected that HSP27 exerted an enormous function on constraining the generation of ROS and the progression of atherosclerosis by inhibiting the mitochondrial apoptosis pathway of CHD (H. L. Zhang et al., 2019). Our experimental results also attested this perspective. The knockdown of HSP27 would lead to excessive production of ROS, and reduce the antioxidant capacity of cells after UVB irradiation. On the basis of this, we deduced that HSP27 could play a photoprotective role by inhibiting the oxidative stress response.

Autophagy is essentially a natural process of self-renewal and self-protection in organism. With the help of the lysosome, damaged, senescent and dying organelles and cytoplasm are devoured, degraded and recycled to balance the intracellular environment. When the cell metabolism or stimulation increases, autophagy will be activated, and the oxidative stress is one of its powerful factors (Wollert, 2019). The p62 protein encoded by *SQSTM1* gene, which not only plays an indispensable role in autophagy, but also an effective target gene of the antioxidant system. For autophagy, p62 operates through the ubiquitin-proteasome pathway, binding to ubiquitinated products and targeting and transporting them to the autophagosome. While binding to lysosomes, the autophagosome contents, including p62, will be degraded (Kumsta et al., 2019). In terms of antioxidant, p62 is better known as a natural activator of Nrf2-antioxidant response element (ARE) transcription pathway. p62 binds to kelch-like ech associated protein 1 (Keap1), leading to dissociation of Keap1 and Nrf2. Then Nrf2 is released and accumulated in the cytoplasm, promoting its binding to the specific sequence ARE. And then a series of the downstream genes were activated to regulating the expression of several antioxidant enzymes including SOD1, SOD2 and CAT. (Bartolini et al., 2018). In addition, recent studies have found that under the induction of PPARGC1A, p62 had anti-aging effect by activating autophagy and upregulating the expression of antioxidant proteins. (Salazar et al., 2020). In this study, autophagy was up-regulated, and the antioxidant system was also activated by UVB radiation. Whether p62 or other antioxidant pathways serve as a bridge between these two events will be one of the focuses of our future research.

Taken together, these findings demonstrated that HSP27 might protect cells or tissues from the adverse effects of UVB irradiation by stimulating autophagy and reducing ROS production, offering proofs that HSP27 might be an effective therapeutic target for the prevention of UVB irradiation-induced skin lesions. Regrettably, we have not yet determined the specific pathways by which HSP27 regulates autophagy and ROS levels to exert its anti-photodamage function, and the existence of correlation between UVB-induced autophagy activation and the occurrence of oxidative stress. In future studies, we will make efforts to clarify the definite mechanism of HSP27 photoprotection.

## Data Availability

The original contributions presented in the study are included in the article/Supplementary Material, further inquiries can be directed to the corresponding authors.
